# Supplementary material for: HIF1A Knockout by Biallelic and Selection-Free CRISPR Gene Editing in Human Primary Endothelial Cells with Ribonucleoprotein Complexes
Source: Biomolecules. 2022 Dec 22;13(1):23. doi: 10.3390/biom13010023 (PMC9856017; doi:10.3390/biom13010023)
Supplement: Supplementary file 1 [file biomolecules-13-00023-s001.zip › biomolecules-2067498-supplementary figures.pdf]

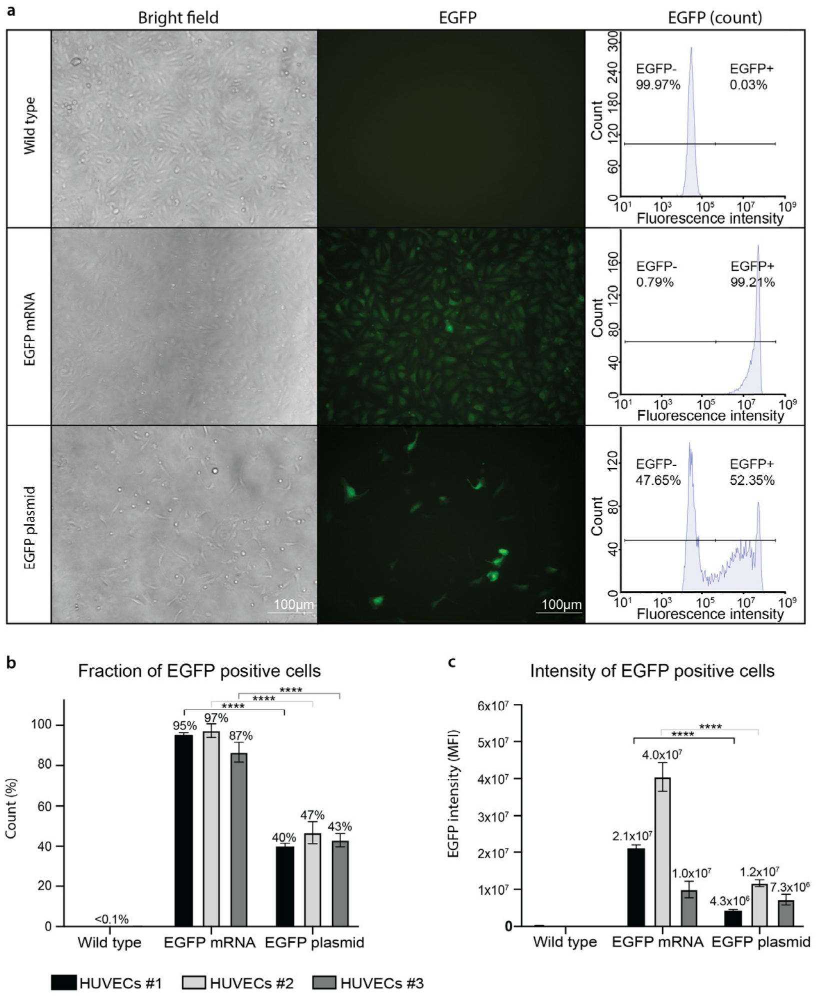

Figure S1: Nucleofection of HUVECs with EGFP mRNA or EGFP plasmid. **S1a:** Visualization and quantification of the EGFP positive HUVECs by fluorescent- and bright field and FC analysis. FC was used to determine the fraction of EGFP-positive cells. **S1b:** The fraction of EGFP-positive HUVECs at each EGFP mRNA concentration. Values are presented as the mean percentage of EGFP-positive cells ±STD. The EGFP mRNA and EGFP plasmid nucleofected HUVECs are compared by one-way ANOVA with Tukey multiple comparisons test, * $p \leq 0.05$, ** $p \leq 0.01$, *** $p \leq 0.001$, **** $p \leq 0.0001$. **S1c:** Intensity of EGFP-positive HUVECs after nucleofection with EGFP mRNA or EGFP plasmid**.** Values are presented as the MFI of EGFP-positive cells ±SD, which are compared by one-way ANOVA with Tukey multiple comparisons test, * $p \leq 0.05$, ** $p \leq 0.01$, *** $p \leq 0.001$, **** $p \leq 0.0001$. **S1a-c:** Experiments on HUVECs from three biological donors at p. 2-5 (n = 3).

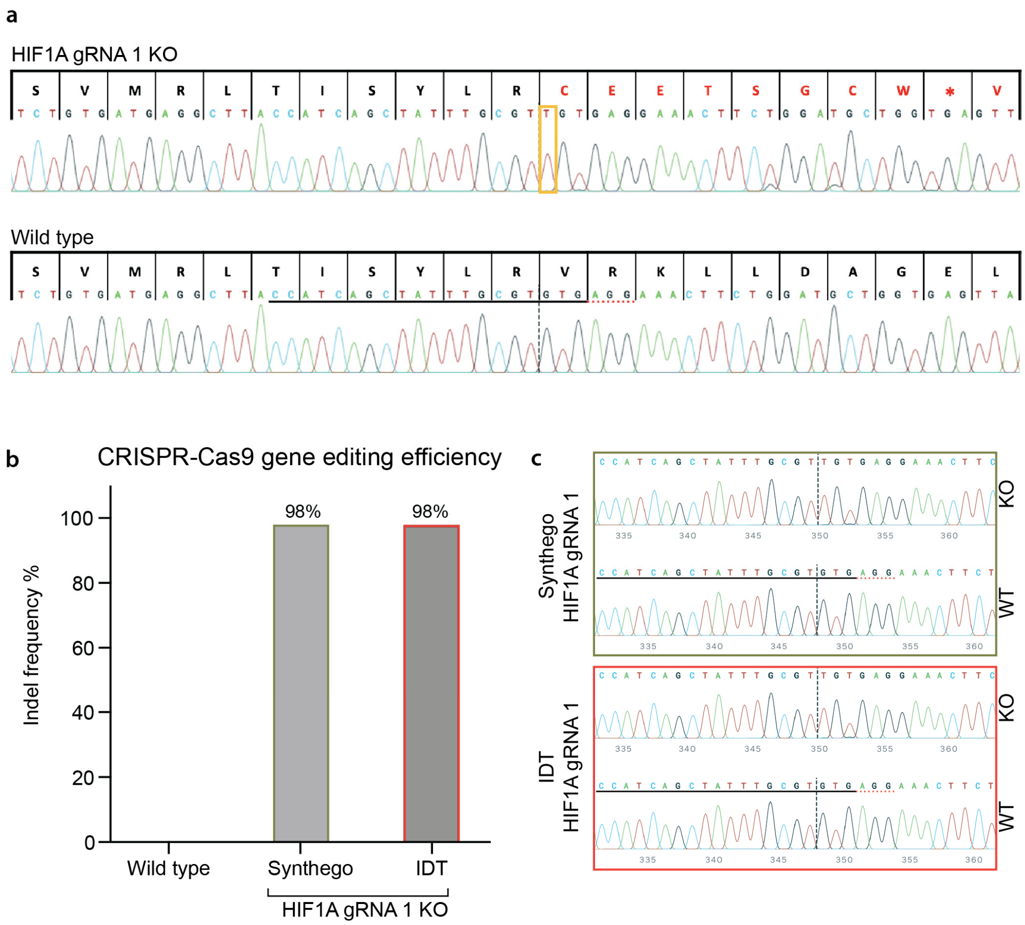

Figure S2: RNP-mediated CRISPR gene editing of HUVECs with HIF1A gRNA 1. **S2a:** Identification of first stop codon after gene editing with HIF1A gRNA 1. The Sanger sequencing results show the nucleotide changes in the HIF1A gRNA 1 gene edited HUVECs versus the WT HUVECs. The black line shows the location of the gRNA and the red dotted line show the location of the PAM sequence. The gene edited nucleotide, thymine, is indicated by the yellow box. The translation of nucleotides to amino acids is shown by the single letter symbols of the amino acids. The first stop codon, TGA, is indicated by *. **S2b:** HIF1A gRNA 1 editing robustness. Quantification of the CRISPR gene editing efficiency of the HIF1A gRNA 1, purchased from two different vendors (Synthego and IDT), and WT HUVECs. Values are presented as the mean percentage of indels ±STD. **S2c:** Representative visualization of the Sanger sequencing results for each HIF1A gRNA 1. For each HIF1A gRNA, the WT HUVECs sequence is shown in the bottom row. The editing site is marked by the black dotted vertical line. The PAM sequence is underlined in dotted red. The sequence of the gene-edited part is shown by wave plots, the nucleotides are color coded as G = guanine, C = cytosine, T = thymine, and A = adenine. **b-c:** Experiments with HUVECs from one biological donor at p. 2 (n = 3).

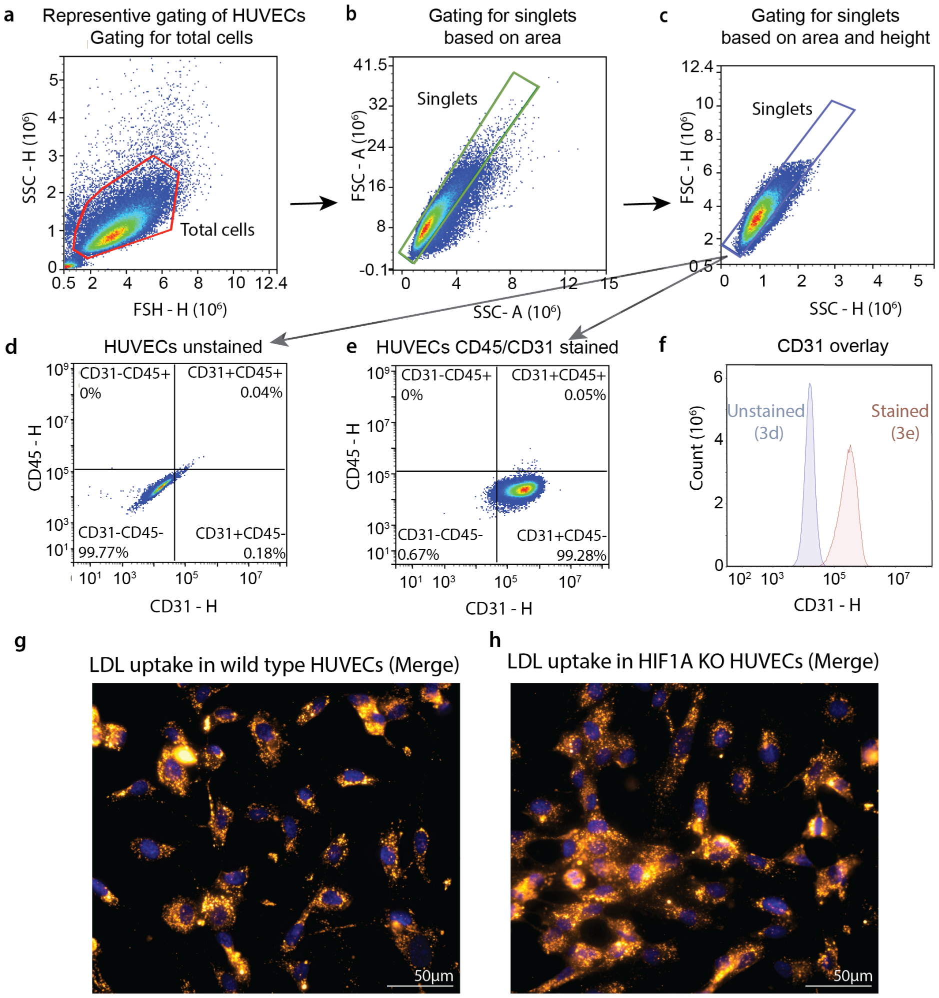

Figure S3: Validation of primary HUVECs. **S3a-f:** Representative gating strategy for FC analysis. **S3a:** FSC-H to SSC-H density plot is used to exclude debris. **S3b-c:** Followed by an SSC-A to FSC-A density plot and an SSC-H to FSC-H plot to exclude doublets. **S3d-e:** After removing the doublets, the relevant cells were visualized in both unstained and CD45/CD31 stained HUVECs. **S3f:** The expression of CD31 was visualized by overlaying the unstained and stained HUVECs plots. FC analyses were done in NovocyteExpress v. 1.5.6. **S3g-h:** LDL uptake in HIF1A KO and WT HUVECs. This figure shows one representative image of the Alexa Fluor 594 LDL treated WT HUVECs, **g**, and HIF1A KO HUVECs, **h**. The HUVECs were treated with 10μg/mL Alexa Fluor 594 LDL (orange) for 4 hours and stained with Hoechst (blue). Scale bar 50μm.

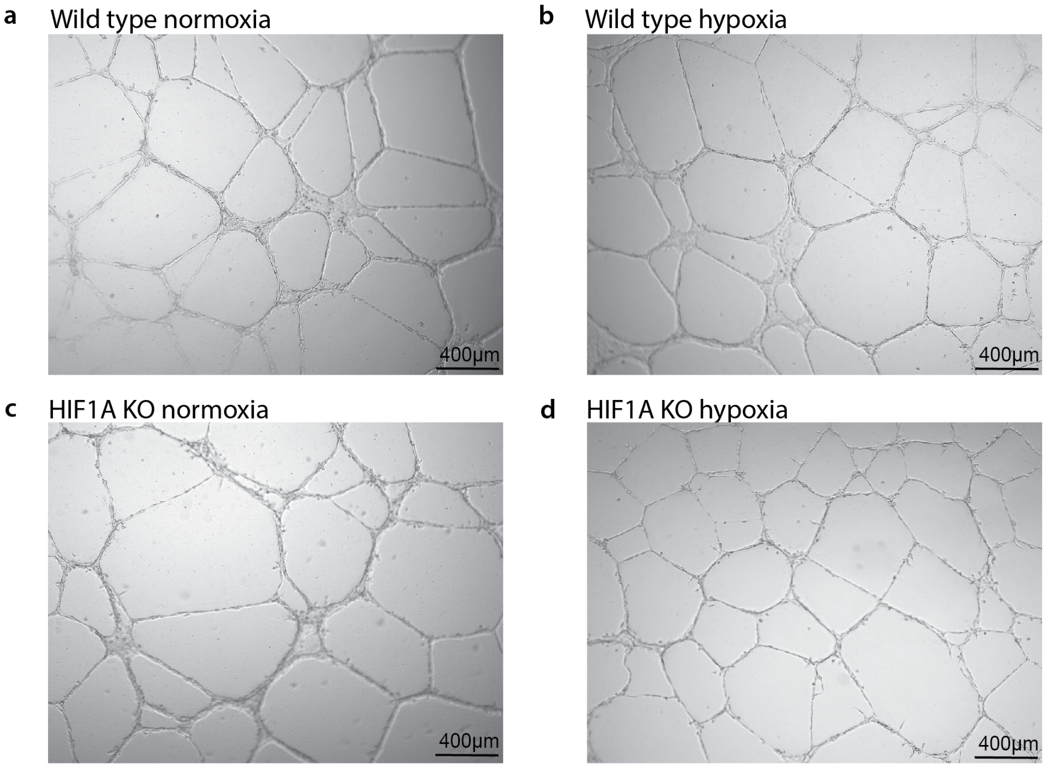

Figure S4: Tube formation assay of HIF1A KO and WT HUVECs. **S4a-d:** Representative bright field images of the TFA HIF1A KO and WT HUVECs cultured in normoxic or 2 hours hypoxic conditions (n = 3). Scale bar 400µm. **S4a-d:** Experiments on HUVECs from one biological donor at final p. 3.

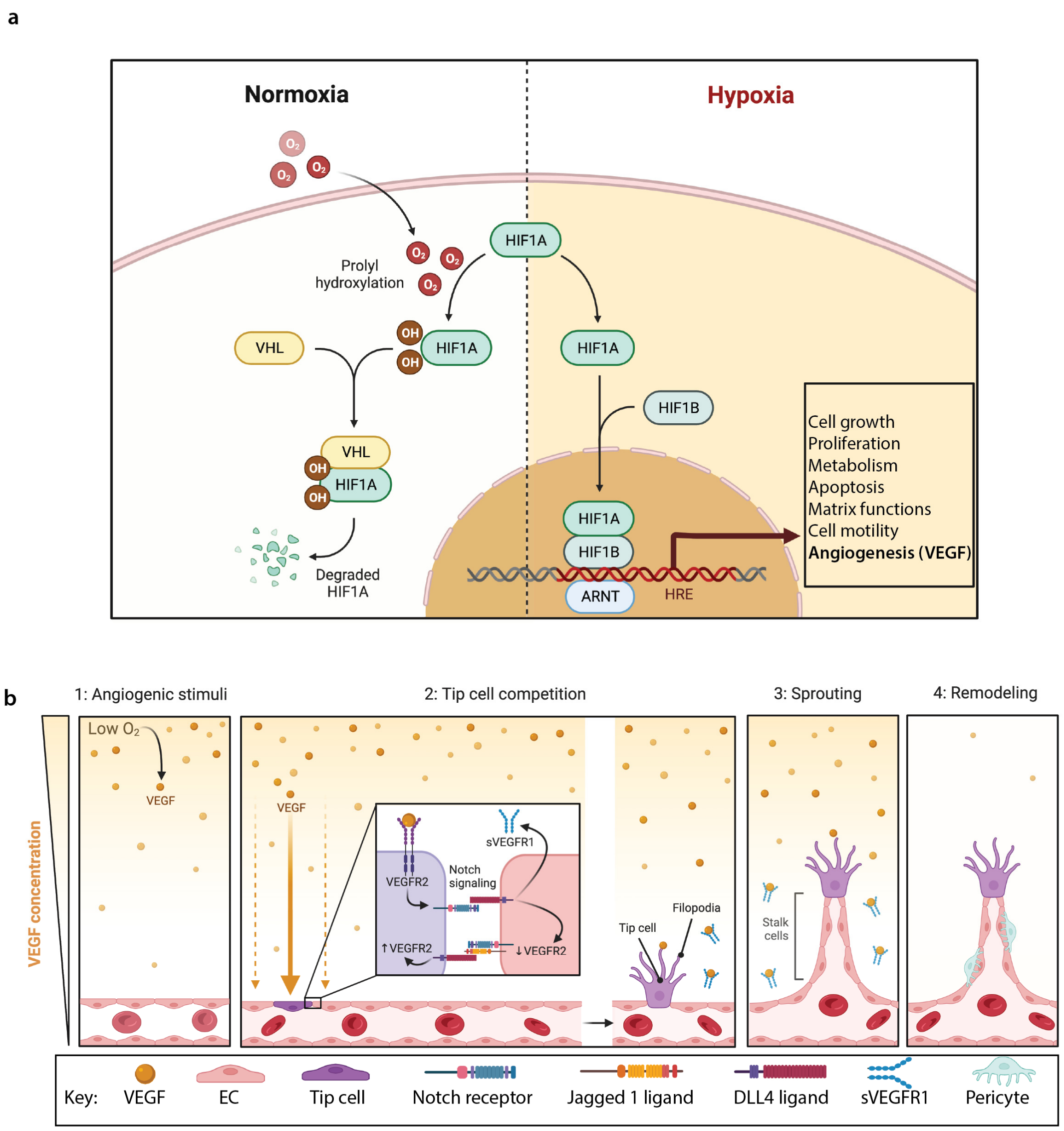

Figure S5: Angiogenesis regulated by hypoxia and VEGF. **S5a:** *HIF1A* pathway in normoxic and hypoxic conditions. In normoxic conditions, HIF1A is prolyl hydroxylated. This allows the binding of von-Hippel Lindau tumor suppressor protein (VHL), which mediates the degradation of *HIF1A*. In hypoxic conditions hydroxylation of HIF1A cannot be facilitated. This leads to translocation of the HIF1A to the nucleus, where it heterodimerizes with the HIF1B and ARNT complex, which initiates the transcription. *HIF1A* is involved in the transcription of over 40 genes, which affect the listed molecular processes. **S5b:** Angiogenesis introduced by VEGF. **1:** The concentration of VEGF increases in hypoxic conditions, by the *HIF1A* mechanism. **2:** The ECs create a tip cell, by VEGF/Notch signaling. VEGF binds to the VEGF receptor 2 (VEGFR2), which upregulates the expression of *delta-like ligand 4* (DLL4) in the tip cell. The tip cell activates Notch signaling in the stalk cells, reducing VEGFR2 in the stalk cells and increases the amount of *soluble VEGFR1* (sVEGFR1). The sVEGFR1 binds the VEGF near the stalk cells, which together with the filopodia and lamellipodia ensures correct orientation of the sprouting. Additionally, the jagged 1 ligand, on the stalk cells, antagonizes the DLL4/notch signaling, which ensures upregulated VEGFR2 expression on the tip cells. **3:** When the tip cell is selected, the stalk cells start to proliferate and form the lumen of the new vessel. **4:** When the sprouting ECs reach the hypoxic area in the tissue, oxygen is delivered to the area. The concentration of oxygen increases, HIF1A is prolyl hydroxylated, and the transcription of VEGF stops. The final step of the vessel formation is the

remodeling phase, where the newly formed vessel matures and stabilizes by the binding of pericytes. The figures are generated with BioRender.com.
